# No Pedigree, No Problem: Genomic Inbreeding Tracks Genetic Rescue With High Resolution

**DOI:** 10.1111/eva.70216

**Published:** 2026-03-11

**Authors:** Carson Mitchell, Samuel Deakin, Marco Festa‐Bianchet, Fanie Pelletier, David Coltman

**Affiliations:** ^1^ Department of Biological Sciences Western University London Ontario Canada; ^2^ Department of Biological Sciences University of Alberta Edmonton Alberta Canada; ^3^ Département de Biologie Université de Sherbrooke Sherbrooke Quebec Canada

## Abstract

With increasing habitat fragmentation and population isolation, inbreeding becomes a pressing concern for the persistence of wildlife populations. Detailed inbreeding monitoring is crucial for assessing extinction risk and evaluating the effectiveness of conservation management strategies. Traditionally, pedigree‐based inbreeding estimates have been used. Genomic approaches now provide more powerful alternatives. Here, we compare pedigree and genomic inbreeding estimates in a long‐term study of wild bighorn sheep ( *Ovis canadensis* ) from Ram Mountain, Alberta, Canada, monitored from 1972 to the present. This population experienced a severe population bottleneck followed by genetic rescue through the translocation of 35 individuals over 13 years. We found that genomic inbreeding coefficients (*F*_ROH_) dropped by 24% after genetic rescue efforts began. In contrast, pedigree inbreeding coefficients (*F*_PED_) increased, likely because greater pedigree depth improved our ability to detect inbreeding in later cohorts, highlighting a methodological bias and the need for genomic monitoring. Our findings show that genomic approaches are more effective in detecting changes in inbreeding over time in wild animals and emphasize the utility of *F*_ROH_ for monitoring the genetic outcomes of conservation interventions, particularly where pedigree completeness increases through time.

## 1 Introduction

Inbreeding (mating among relatives) is a primary concern in conservation because it can reduce survival, reproduction, and genetic diversity, threatening population growth and adaptive potential (Frankham et al. 2002; Hedrick and Garcia‐Dorado 2016). Habitat loss and fragmentation are accelerating population decline and isolation (IPBES 2019), increasing the likelihood of inbreeding and elevating homozygosity (Frankham et al. 2002). This rise in homozygosity can expose previously concealed recessive deleterious alleles, resulting in fitness consequences known as inbreeding depression (Frankham et al. 2002). Because inbreeding can accelerate population decline and increase extinction risk (Lacy 1997; Brook et al. 2002), accurate estimation of inbreeding is essential to assess extinction risk, inform conservation strategies, and evaluate the effectiveness of management interventions (Brook et al. 2002).

One strategy to mitigate inbreeding depression is genetic rescue, which involves introducing unrelated individuals into an inbred population to reduce homozygosity and increase population fitness (Whiteley et al. 2015). If the resulting offspring exhibit increased fitness or if population growth exceeds that expected from demographic supplementation alone, the intervention is considered a successful genetic rescue (Tallmon et al. 2004; Whiteley et al. 2015). Genetic rescue is becoming more common in conservation management, with successful examples in many large mammals, including Florida panthers ( *Puma concolor* ) (Johnson et al. 2010), Mexican grey wolves ( *Canis lupus* ) (Fredrickson et al. 2007), and bighorn sheep ( *Ovis canadensis* ) (Hogg et al. 2006; Miller et al. 2012; Poirier et al. 2019). Genetic rescue often improves population health, growth rate (Whiteley et al. 2015), genetic diversity, and fitness (Clarke et al. 2024), but its effects can weaken over time (e.g., Lotsander et al. 2021). Determining whether genetic rescue leads to sustained improvements in genetic health requires detailed monitoring of inbreeding levels, as well as fitness components such as survival, reproduction, and population growth for several years after release of unrelated individuals.

Researchers quantify inbreeding by measuring the proportion of an individual's genome that is autozygous (*F*), meaning composed of identical copies of DNA that originated in a recent common ancestor (Wright 1922). Traditionally, inbreeding has been estimated using pedigrees (*F*_PED_) (Wright 1922; Pemberton 2004). However, pedigree estimates require detailed family records, which are lacking in most wild populations (Marshall et al. 2002). Incomplete or shallow pedigrees may fail to capture instances of shared ancestry, leading to the incorrect assumption of unrelatedness among mating pairs (Marshall et al. 2002). Missing information can lead to systematic underestimation of pedigree inbreeding. Additionally, *F*_PED_ assumes equal contributions from shared ancestry and does not account for the stochastic nature of inheritance, such that two individuals with the same pedigree can have different levels of autozygosity that *F*_PED_ cannot detect (Kardos et al. 2015).

In contrast, genomic tools now allow for direct estimation of individual‐level autozygosity, even without pedigrees (see Kardos et al. 2023 for an example). One widely used approach involves quantifying runs of homozygosity (ROH)—contiguous homozygous regions inherited from common ancestors (Shafer and Kardos 2024). When parents are related, their offspring are more likely to inherit identical haplotypes from both parents, resulting in long stretches of homozygosity. By sampling the genome for homozygous alleles, we can estimate the location and length of ROH. The proportion of the genome contained in ROH (*F*_ROH_) serves as a genomic estimate of inbreeding, with longer ROH reflecting more recent shared ancestry and typically harboring the recessive deleterious alleles responsible for inbreeding depression (Pemberton et al. 2012; Szpiech et al. 2013; Kyriazis et al. 2025).

Simulations have revealed that *F*_ROH_, when based on a large number of single‐nucleotide polymorphisms (SNPs), reflects autozygosity more accurately than *F*_PED_, outperforming pedigrees that are fully complete to 20 generations (Kardos et al. 2015). Unlike pedigree inbreeding coefficients, genomic inbreeding coefficients can capture individual variation in autozygosity, making them more sensitive for detecting the fitness consequences of inbreeding (Huisman et al. 2016; Nishio et al. 2023). In red deer ( *Cervus elaphus* ), genomic metrics allowed Huisman et al. (2016) to detect inbreeding depression in adult and juvenile traits, whereas pedigree inbreeding only revealed it in juvenile traits. As sequencing technologies become more accessible, a growing consensus is emerging that genomic inbreeding estimates should replace pedigree‐based metrics in conservation genetics (Kardos et al. 2015; Wang 2016; Peripolli et al. 2017; Brüniche‐Olsen et al. 2018). Despite widespread advocacy for genomic monitoring (e.g., Kardos et al. 2015, 2016; Huisman et al. 2016; Ashraf et al. 2022), and a report that *F*_PED_ consistently underestimated inbreeding relative to *F*_ROH_ in a highly inbred population with a successful immigrant (Robinson et al. 2019), it remains unclear how *F*_PED_ and *F*_ROH_ compare in monitoring a genetic rescue in a wild population.

This study aimed to compare pedigree and genomic estimates of inbreeding following a genetic rescue of a small wild population of bighorn sheep, where nearly all individuals have been monitored since 1972 (Festa‐Bianchet et al. 2019). Pre‐rescue pedigree inbreeding has been explored in this population (Rioux‐Paquette et al. 2011), but another investigation is warranted given the emergence of genomic tools and recent population dynamics following an attempted rescue. We calculated pedigree inbreeding (*F*_PED_) from long‐term parentage records and genomic inbreeding (*F*_ROH_) from SNP data. We then evaluated trends in pedigree and genomic inbreeding across a severe population decline and subsequent genetic rescue through artificial supplementation (Poirier et al. 2019). We hypothesized that pedigree and genomic inbreeding coefficients would respond to genetic rescue, but not to the same extent. We predicted that inbreeding would decrease after translocations, but that *F*_ROH_ would exceed *F*_PED_ and exhibit a greater reduction in response to genetic rescue.

## 2 Methods

### 2.1 Study Site and Population Dynamics

Ram Mountain (52° N, 115° W; elevation 1080–2170 m; area ≈38 km^2^) is home to a small, isolated population of Rocky Mountain bighorn sheep monitored since 1972 (Jorgenson et al. 1993). The population, located 30 km from the main Canadian Rocky Mountain range in Alberta, Canada, is isolated by forest, an unsuitable habitat for bighorn sheep (Festa‐Bianchet et al. 2019), and is genetically distant from populations in the main range (Deakin et al. 2020). Bighorn sheep live in sexually segregated groups outside the autumn rut (Pelletier and Festa‐Bianchet 2006), with lambs born roughly 6 months later (Hogg et al. 2017). Reproductive contributions peak between ages 3 and 12 for ewes and increase with age for rams (Coltman et al. 2002; Festa‐Bianchet 2012; Festa‐Bianchet et al. 2019).

Since 1975, over 99% of yearling and adult sheep in the study population have been individually marked. From May to October, about 90% of the population is recaptured in a corral trap baited with salt (Coltman et al. 2002; Festa‐Bianchet et al. 2019; Poirier et al. 2019). Since 1987, when DNA sampling started, biological samples were collected at the time of capture. Hair samples were stored in paper envelopes or plastic bags with 5 g of silica at room temperature, and tissue samples were stored in a 20% dimethyl sulphoxide‐saturated NaCl solution at −20°C (Coltman et al. 2002). Not all sample types were collected every year.

**FIGURE 1:**
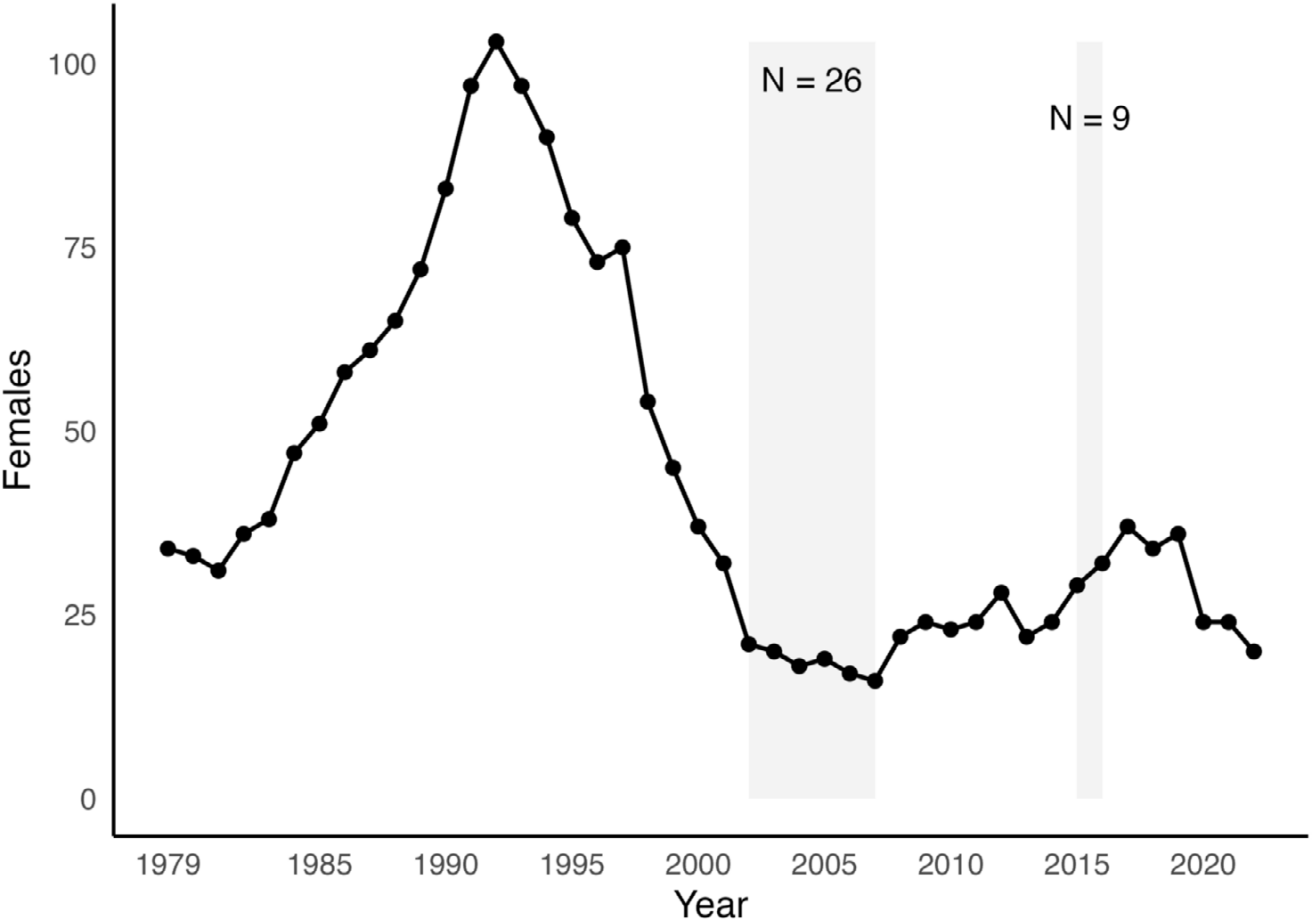
Number of ewes aged 2 and older from Ram Mountain, Alberta, Canada, between 1979 and 2022. Shaded areas indicate years during which individuals were translocated to the population as part of a genetic rescue effort with the number of translocated individuals labeled in each shaded zone.

The Ram Mountain population has undergone substantial demographic shifts over the past five decades (Figure 1). In early years, annual experimental removals limited the number of adult females to ~30 (Festa‐Bianchet et al. 2019). Following the cessation of removals in 1980, the population increased rapidly, peaking in 1992. It subsequently declined by 83% due to density‐dependent effects (Festa‐Bianchet et al. 2003) and predation by cougars ( *P. concolor* ) (Festa‐Bianchet et al. 2006), experiencing a population bottleneck (Poirier et al. 2019). Concerns about inbreeding prompted a genetic rescue effort (Rioux‐Paquette et al. 2011).

Between 2002 and 2007, 26 bighorn sheep of mixed age and sex were translocated from the Cadomin area (approx. 130 km Northwest) via truck and helicopter in late winter to support population recovery (Poirier et al. 2019). After a secondary population decline linked to continued predation, a second translocation of nine individuals occurred in 2015 (Poirier and Festa‐Bianchet 2018). Confirmation of a successful genetic rescue came when first‐ and second‐generation hybrid lambs weighed more and had higher survival compared to lambs with endemic ancestry (Poirier et al. 2019).

### 2.2 Pedigree Inbreeding ( *F*_PED_ )

A multigenerational pedigree was constructed using field observations and microsatellite genotyping of hair and tissue samples (Coltman et al. 2002, 2005). Maternal links were established through suckling observations, while paternal assignments and confirmation of maternal identity were based on ~30 microsatellite loci typed via PCR amplification and electrophoresis as described by Coltman et al. (2002, 2005). Parentage was assigned in CERVUS v3.0 (Marshall et al. 1998; Kalinowski et al. 2007) at 95% confidence using a likelihood approach.

We calculated pedigree inbreeding coefficients (*F*_PED_) using the *ip_F*() function from the purgeR package (López‐Cortegano 2022). *F*_PED_ quantifies the probability that an individual inherits identical alleles from both parents due to shared ancestry and theoretically ranges from 0 (no inbreeding) to 1 (complete inbreeding) (Wright 1922; Malécot 1948). Coefficients were derived using the standard recursive tabular algorithm based on the additive relationship matrix, in which an individual's inbreeding coefficient equals the kinship between its parents. Values were manually verified for a subset of individuals to confirm accuracy.

Pedigrees in wild populations often contain gaps that limit detection of nonzero inbreeding coefficients (Marshall et al. 2002). In bighorn sheep, maternal links are readily identified because suckling can be observed. In contrast, paternal links must be inferred genetically, since rams provide no parental care. Microsatellite genotyping was unavailable during the first 15 years of the Ram Mountain study, resulting in incomplete paternity data. Across the full pedigree (*n* = 1262), maternal identity was known for 78% of individuals (*n* = 982) and paternal identity for 51% (*n* = 642). Individuals with unknown parents were assigned *F*_PED_ = 0 but excluded from analyses to reduce the underestimation of inbreeding, unless they were translocated or a direct offspring of a translocated sheep, scenarios in which we can be more confident that individuals are truly outbred. Marshall et al. (2002) showed that at least one known grandparent is required to detect *F*_PED_ = 0.125 (the expectation for half‐sibling or grandparent‐offspring matings); accordingly, prior studies (e.g., Kruuk et al. 2002; Szulkin et al. 2007; Rioux‐Paquette et al. 2011) typically retained individuals with ≥ 1 grandparent known. We adopted a stricter criterion—requiring all four grandparents to be identified—to improve detection of a broader range of inbreeding pathways and increase confidence in *F*_PED_ estimates, while retaining a representative sample. Although detecting finer‐scale inbreeding would require deeper pedigrees, only 64 individuals (5%) had complete great‐grandparental information. Restricting analyses to these individuals would reduce our dataset by 195 individuals, biasing it toward more recent cohorts and overrepresenting translocated sheep and their direct descendants, while substantially reducing statistical power. We therefore restricted analyses to 259 individuals with complete grandparental information, except when *F*_PED_ was nonzero (*n* = 19) or the individual was translocated from Cadomin or an offspring of a Cadomin immigrant (*n* = 86). After filtering, we retained 364 individuals (29%) for analysis of *F*_PED_. This filtering may bias results by overrepresenting well‐documented lineages and excluding truly outbred individuals with shallow pedigrees.

### 2.3 Genotyping and SNP Filtering

We genotyped 384 individuals using a 50,000 SNP Allegro Targeted Genotyping assay described in Deakin and Coltman (2024) and Deakin (2023). DNA was extracted from tissue samples using the DNeasy Blood and Tissue Kit (Qiagen, Venlo, Netherlands), and genotyping libraries were prepared using the Allegro Targeted Genotyping V1 kit (Tecan Genomics, Redwood City, CA, USA). Sequencing was performed on an Illumina NextSeq v2.5 platform (San Diego, CA, USA). We obtained the raw sequencing reads from Deakin (2023) and proceeded with the following bioinformatics pipeline.

We performed initial quality control of raw sequence reads using FastQC (Andrews 2010) and concatenated lane‐specific reads. We conducted adapter trimming and quality filtering using Trimmomatic v0.39 (Bolger et al. 2014) with Phred+33 encoding. Next, we aligned reads to a bighorn sheep reference genome (GenBank Accession: JAJQZS000000000) using BWA v0.7.18 (Li and Durbin 2009). We then converted SAM files to BAM format and sorted and indexed them using SAMtools v1.20 (Li et al. 2009). We performed variant calling with BCFtools v1.19 (Li et al. 2009), yielding ~9 million SNPs.

We filtered variant sites to retain high‐quality, informative SNPs using VCFtools v0.1.16 (Danecek et al. 2011). We excluded indels and non‐biallelic sites to reduce genotyping error and simplify downstream analyses. We applied additional filters to remove SNPs with low quality (< 30), low depth (< 20), high missingness (> 10%), low minor allele count (< 8), and deviations from Hardy–Weinberg equilibrium (*p* < 0.01), in that order. We also excluded individuals with >25% missing data and technical replicates. To reduce linkage disequilibrium, SNPs were thinned based on physical distance, retaining only one site per 100 bp window. After filtering, 38,731 SNPs remained, corresponding to an average density of ~14.7 SNPs per Mb across the 2.63 Gb reference genome.

### 2.4 Genomic Inbreeding

We estimated genomic inbreeding coefficients (*F*_ROH_) from runs of homozygosity (ROH), defined as contiguous homozygous segments arising from shared ancestry. We identified ROH using the *roh* command in BCFtools, which applies a hidden Markov model that probabilistically assigns genomic regions to autozygous or non‐autozygous using genotypes or genotype likelihoods and allele frequencies (Narasimhan et al. 2016). We used genotypes with the ‐G30 option, corresponding to genotype quality, to reduce low‐confidence contributions to ROH inference and provided allele frequencies by tagging the filtered VCF prior to ROH identification (Narasimhan et al. 2016). The constant default recombination rate and default transition probabilities were used. BCFtools requires fewer user‐defined parameters, such as length thresholds, compared to other ROH‐detection software, which can reduce the subjectivity of ROH inference, while maintaining high accuracy (Narasimhan et al. 2016). Still, we excluded extremely short ROH (< 1 Mb) from our dataset, since they are likely derived from ancient inbreeding (Pemberton et al. 2012) and are less relevant to inbreeding depression (Szpiech et al. 2013; Kyriazis et al. 2025). We calculated *F*_ROH_ in R v4.4.3 as *F*_ROH_ = ΣL_ROH_/ΣL_auto_, where ΣL_ROH_ is the total length of ROH and ΣL_auto_ is 2.46 Gb, based on the autosomal genome size.

We calculated individual genome‐wide expected and observed homozygosity and F_IS_ with VCFtools v0.1.16 (‐‐het). Two individuals were identified as outliers and excluded from genome‐wide assessments of observed homozygosity and *F*_IS_ due to SNP ascertainment bias; the custom SNP panel was designed to target some of the variants detected in the whole genome sequence of one of these individuals (Miller et al. 2015; Deakin and Coltman 2024), enriching the dataset for loci at which this individual is heterozygous. Both this individual and its parent therefore showed inflated heterozygosity and strongly negative F_IS_ values that would bias genome‐wide estimates. Both individuals were retained for ROH analyses because ROH depend on extended homozygous tracts and are substantially less sensitive to SNP ascertainment bias. A third outlier was retained, as the pattern is biologically plausible and likely reflects high parental divergence.

High‐quality genomic data with sufficient marker density were available for 320 individuals born between 1979 and 2017 and were used for ROH‐based inbreeding analyses. After excluding two individuals affected by SNP ascertainment bias, 318 were retained for genome‐wide *F*_IS_ and homozygosity estimates. Of the 320 individuals in the total genomic sampling pool, 146 overlapped with the filtered pedigree dataset for paired *F*_ROH_–*F*_PED_ comparisons.

### 2.5 Direct Comparison of *F*_PED_ and *F*_ROH_

We used a Wilcoxon signed‐rank test to compare individual inbreeding coefficients derived from pedigrees (*F*_PED_) and genomic data (*F*_ROH_) for individuals with both estimates available (*n* = 146). To assess the association between *F*_PED_ and *F*_ROH,>1Mb_, we performed Kendall's tau correlation, appropriate for nonparametric data with tied values due to the prevalence of zero *F*_PED_ estimates.

### 2.6 Inbreeding Over Time

We explored temporal trends in inbreeding by modeling *F*_ROH,>1Mb_ (*n* = 320) and *F*_PED_ (*n* = 364) as a function of birth year (or year of introduction for the translocated individuals) using beta‐regression models implemented with glmTMB (Brooks et al. 2017). Genomic data were available for sheep born between 1979 and 2017 and pedigree data for sheep born between 1987 and 2022. A beta distribution is appropriate because *F* represents the probability of identity‐by‐descent and is therefore naturally bounded between 0 and 1. We adjusted 0 *F*_PED_ values to < 0.001 to avoid boundary issues during model fitting and because no individual is expected to be entirely devoid of autozygosity (Keller et al. 2011). We applied natural cubic splines with three degrees of freedom to capture potential nonlinear changes in inbreeding corresponding to the three main demographic phases: population expansion (1979–1992), decline (1992–2002), and genetic rescue (2002–2022). The primary purpose of this analysis was to visualize broad changes in inbreeding trends over time. We illustrated the models with raw data points for transparency. To understand what lengths of ROH are most impacted by the population decline and rescue, we separated ROH into three length categories: 1–5 Mb, 5–20 Mb, and > 20 Mb. Because recombination progressively shortens IBD segments over time (Thompson 2013), longer ROH are expected to represent more recent inbreeding. We calculated *F*_ROH_ separately for the three length categories and fit beta models for each binned *F*_ROH_ category as a function of year with natural cubic splines (3 df) as described above. We also modeled individual genome‐wide homozygosity (*n* = 318) over time with a beta model with natural cubic splines (3 df) in the same manner, as a complementary measure of genomic diversity.

### 2.7 Inbreeding Before and After Rescue

To assess whether genetic rescue was associated with a shift in inbreeding levels, we fit two beta‐regression mixed models using the glmTMB package (Brooks et al. 2017) with *F*_PED_ (*n* = 364; 0 values adjusted to < 0.001) and *F*_ROH>1Mb_ (*n* = 320) as response variables and population group (before or after rescue) as a fixed effect. We included year as a random intercept to account for non‐independence among individuals born in the same year. Individuals born in or before 2002 were classified as pre‐rescue, and those born after 2002 as post‐rescue. Translocated individuals were assigned to the post‐rescue group regardless of birth year.

### 2.8 Pedigree Depth

As *F*_PED_ values depend on the completeness of pedigree information, we investigated whether pedigree depth differed between pre‐ and post‐rescue individuals and how depth influenced *F*_PED_ values. We calculated complete generational equivalents (CGE) as the sum of contributions from all known ancestors, weighted by generational distance (∑(0.5)^g^, where g is the number of generations away from the focal individual). We verified the resulting values manually for a subset of individuals to confirm accuracy. We compared the average CGE between pre‐ and post‐rescue population groups using Welch's *t*‐test, including translocated sheep in the post‐rescue group. We then removed individuals with a CGE of 1 or less from our filtered dataset to avoid anchoring the regression on individuals whose *F*_PED_ is constrained to zero. We evaluated the trend between *F*_PED_ (*n* = 327; 0 values adjusted to < 0.001) and average pedigree depth using a beta‐regression model, with year included as a random effect to account for non‐independence among individuals born in the same year.

We conducted all statistical analyses in R v4.4.3 and used *α* = 0.05, with plotting performed using ggplot2 v3.5.2 (Wickham 2016) and model‐predicted values extracted with ggeffects v2.3.1 (Lüdecke 2018). Model assumptions were evaluated using diagnostic plots.

## 3 Results

### 3.1 Direct Comparison of Inbreeding Coefficients

**FIGURE 2:**
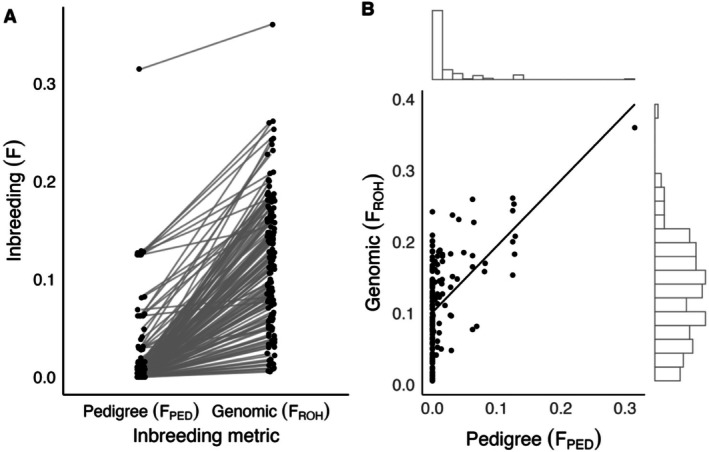
Comparison of pedigree (*F*_PED_) and genomic (*F*_ROH,>1Mb_) inbreeding coefficients for bighorn sheep on Ram Mountain. (A) Paired dot plot showing individual inbreeding values derived from pedigrees and from runs of homozygosity (ROH), with lines connecting matched individuals. (B) Scatterplot with marginal histograms showing the correlation between *F*_PED_ and *F*_ROH,>1Mb_ with a correlation line overlaid.

Genomic inbreeding coefficients (*F*_ROH_) were always higher than pedigree estimates (*F*_PED_) (Wilcoxon signed‐rank test: *V* = 0, *p* < 0.001, *n* = 146; Figure 2A). *F*_PED_ (*n* = 364) ranged from 0 to 0.315 with 215 zero values, a mean of 0.021 and a strong right skew. *F*_ROH,>1Mb_ (*n* = 320) had a similar range of 0.005 to 0.360, a near‐normal distribution and a higher mean of 0.122. The two metrics were positively but weakly correlated (Kendall's Tau: *τ* = 0.382, *p* < 0.001, *n* = 146; Figure 2B), indicating that while they capture similar inbreeding signals, *F*_ROH_ detects inbreeding that *F*_PED_ fails to capture. *F*_ROH,1–5Mb_ ranged from 0.003 to 0.047 and had a mean of 0.025, *F*_ROH,5–20Mb_ ranged from 0 to 0.140 and had a mean of 0.060 and *F*_ROH,>20Mb_ ranged from 0 to 0.191 with a mean of 0.037. Observed genome‐wide homozygosity ranged from 0.544 to 0.765 with a mean of 0.686. Individual F_IS_ ranged from −0.463 to 0.250 with a mean near zero (−0.004), indicating little heterozygosity deviation from Hardy–Weinberg expectations after quality‐control filtering.

### 3.2 Inbreeding Over Time

**FIGURE 3:**
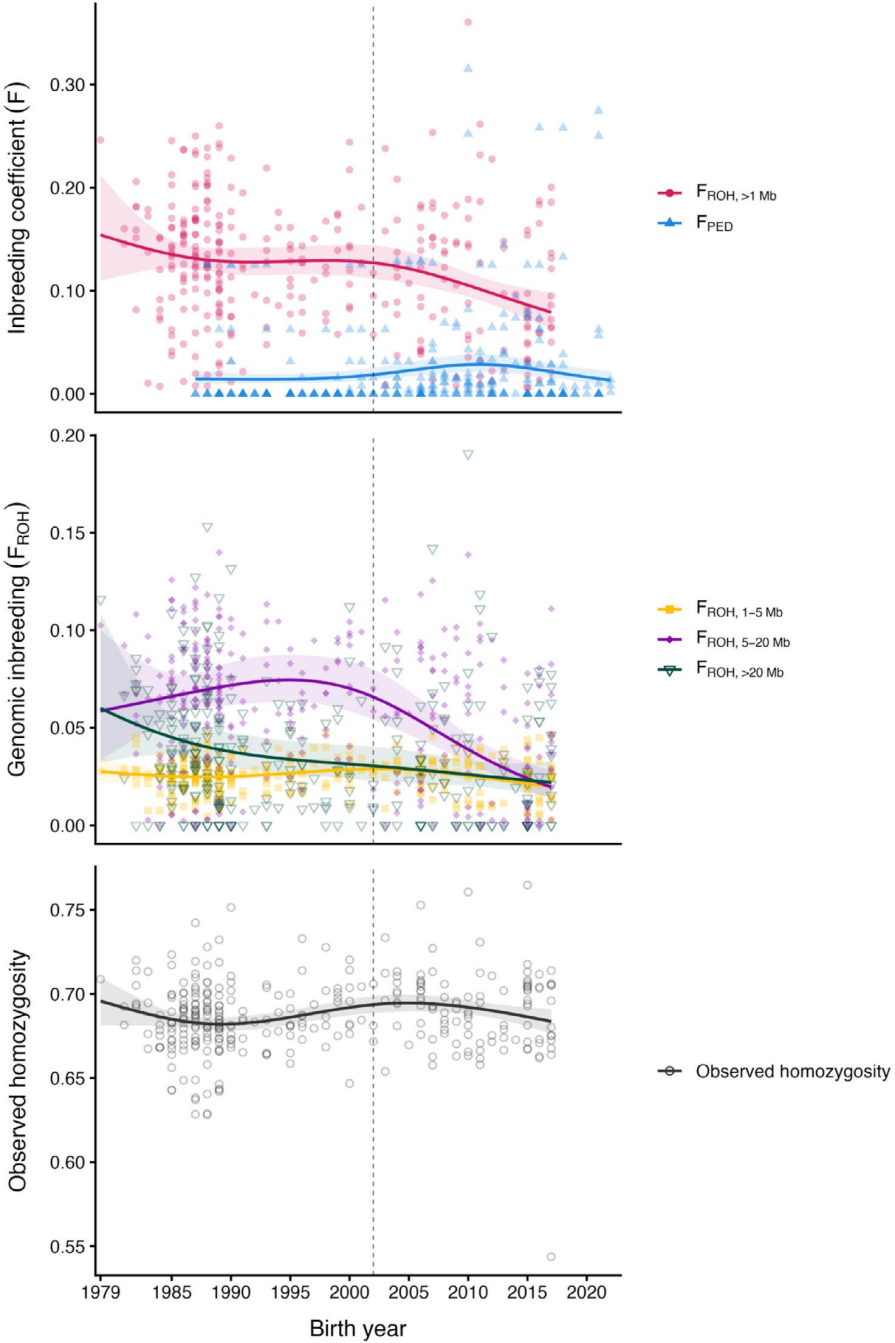
Temporal trends in inbreeding in the Ram Mountain bighorn sheep population, (Alberta, Canada). The top panel shows genomic (*F*_ROH,>1Mb_) and pedigree (*F*_PED_) inbreeding coefficients; the middle panel shows genomic inbreeding coefficients partitioned by ROH length (*F*_ROH,1–5Mb_, *F*_ROH,5–20Mb_, and *F*_ROH,>20Mb_); and the bottom panel shows observed homozygosity. Solid lines show predicted values from beta‐regression models with natural splines (3 df), with 95% confidence intervals shown as shaded regions. Individual data points are overlaid to illustrate variation among individuals. The dashed vertical line in 2002 marks the initiation of genetic rescue efforts. Translocated individuals and their descendants are included in all panels.

We modeled temporal change in *F*_ROH,>1Mb_ (*n* = 320) and *F*_PED_ (*n* = 364), *F*_ROH,1–5Mb_, *F*_ROH,5–20Mb_, and *F*_ROH_ _,>20Mb_ (*n* = 320), and observed genome‐wide homozygosity (*n* = 318) using beta regressions with natural splines (3 df) to allow for nonlinear variation as the population went through a period of expansion, decline and rescue. *F*_ROH,>1Mb_ changed nonlinearly over time, and despite substantial variation, the fitted curve showed a clear decline in genomic inbreeding following the onset of genetic rescue (Figure 3). In contrast, *F*_PED_ did not show a comparable post‐rescue decline (Figure 3).

### 3.3 Inbreeding Before and After the Initiation of Rescue Efforts

**FIGURE 4:**
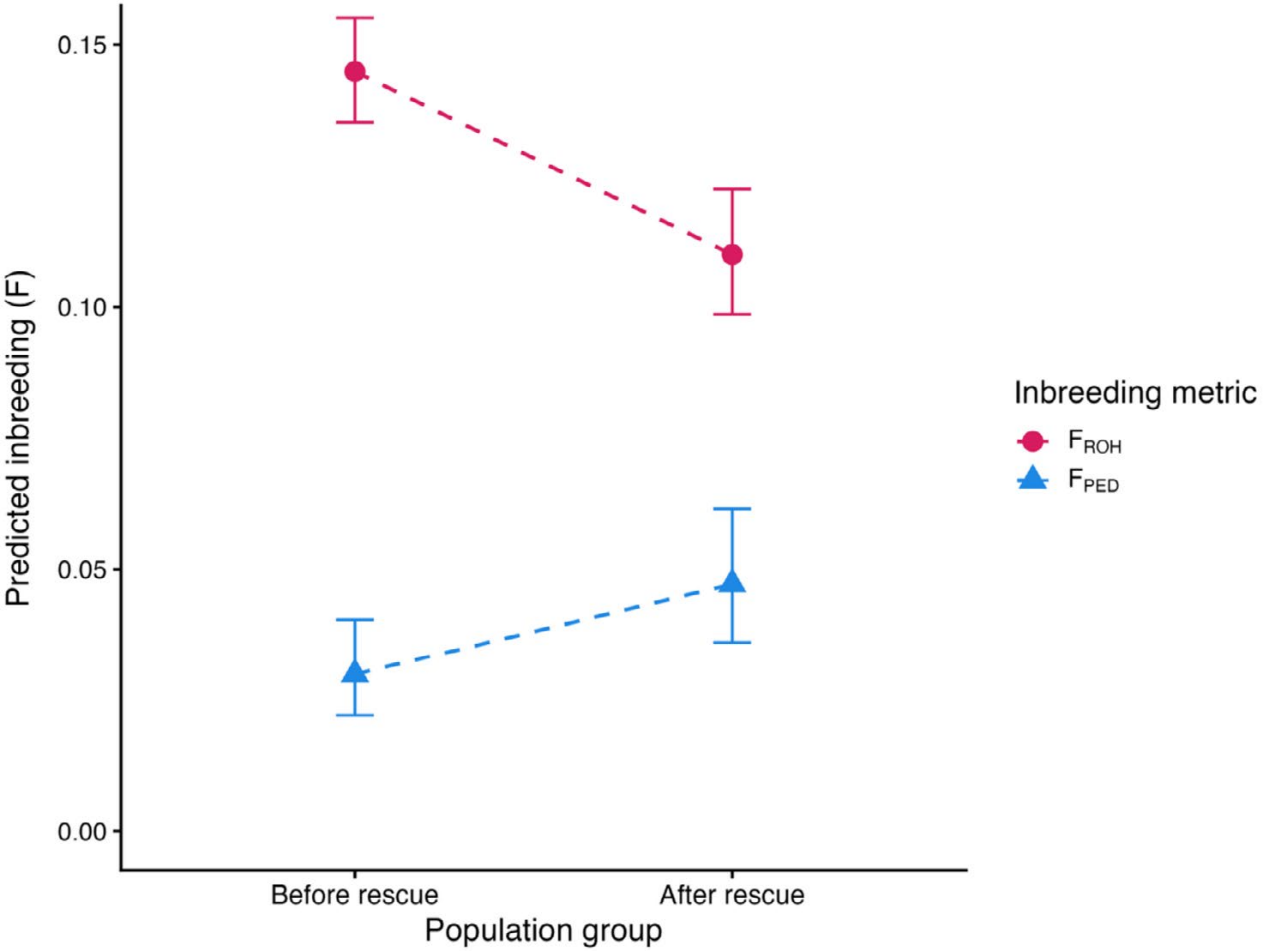
Predicted genomic (*F*_ROH,>1Mb_) and pedigree (*F*_PED_) inbreeding coefficients before and after the initiation of genetic rescue efforts. The *before* group includes individuals born on Ram Mountain in or before 2002, and the *after* group includes individuals born after 2002 as well as translocated individuals. Points show model‐predicted means from beta‐regression models with year as a random intercept, and vertical lines indicate 95% confidence intervals. The dashed line illustrates the magnitude and direction of change between groups.

To test for changes in population‐wide inbreeding levels before and after the rescue event began, we fit beta models for *F*_ROH,>1Mb_ (*n* = 320) and *F*_PED_ (*n* = 364) by population group (before and after rescue) and a random intercept for year. The model predicted that *F*_ROH,>1Mb_ dropped by 24%, from 0.145 to 0.110, between the pre‐ and post‐rescue groups, while *F*_PED_ increased by 58%, from 0.030 to 0.047 (*F*_ROH,>1Mb_: *β* = −0.324 ± 0.074 SE, *z* = −4.37, *p* < 0.001; *F*_PED_: *β* = 0.479 ± 0.109 SE, *z* = 4.41, *p* < 0.001; Figure 4). This creates an apparent contradiction, as *F*_ROH,>1Mb_ and *F*_PED_ show opposing trends.

### 3.4 Resolving the Contradiction

**FIGURE 5:**
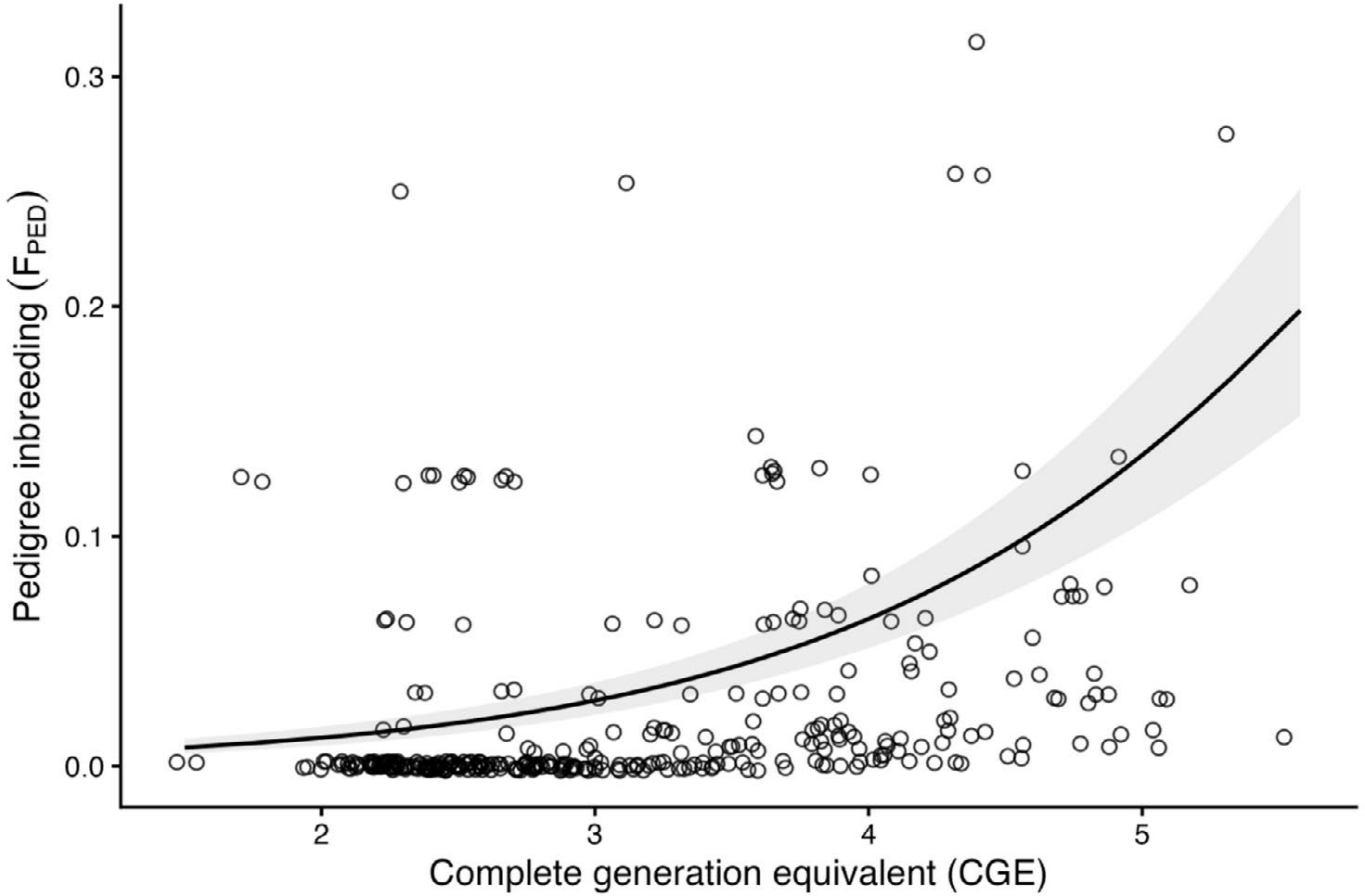
Pedigree inbreeding coefficient (*F*_PED_) by complete generation equivalent (CGE) for Ram Mountain bighorn sheep for individuals with a CGE greater than 1. The solid line represents the predicted values of the beta‐regression model with 95% confidence intervals shown as shaded areas. Points are jittered to better visualize the number of observations.

The drop in *F*_ROH,>1Mb_ post‐rescue reflects reduced inbreeding driven by the contribution of translocated and admixed individuals, which have substantially lower mean *F*_ROH,>1Mb_ values (0.063 and 0.060, respectively) compared to sheep with exclusively endemic ancestry (0.134). These outbred individuals with much lower genomic inbreeding coefficients in the after‐rescue group drove the observed drop. For *F*_PED_, the observed and unexpected positive change is likely caused by the systematic increase in pedigree depth as records expand over time, providing more accurate inbreeding measurements in post‐rescue cohorts, evidenced by greater CGE for the post‐rescue group and a positive trend between CGE and *F*_PED_ (*t*‐test: mean before = 2.58, mean after = 3.03, *t* = −4.28, 95% CI: −0.649 to −0.240, *p* < 0.001, *n* = 364; beta‐regression model: *β* = 0.858 ± 0.0701 SE, *z* = 12.3 *p* < 0.001, *n* = 327; Figure 5).

## 4 Discussion

This long‐term study provides a direct comparison of pedigree and genomic inbreeding coefficients around a genetic rescue event. Our results demonstrate that genomic estimates (*F*_ROH_) outperform pedigree coefficients (*F*_PED_) in detecting recent changes in autozygosity and should be prioritized when evaluating management interventions in the wild.

### 4.1 Genetic Rescue Success

The drop in genomic inbreeding coefficients after rescue efforts began suggests that genetic rescue decreased inbreeding in the Ram Mountain bighorn sheep population (Poirier et al. 2019). Translocations and crosses between immigrants and Ram Mountain residents drove the decline in inbreeding. Crossing facilitated the breakdown of ROH and likely masked the expression of some deleterious recessive alleles, thereby aiding demographic recovery. Consistent with this mechanism, the strongest decline appeared in *F*_ROH,5–20Mb_, representing intermediate to long ROH, typically originating from recent shared ancestry that may have accumulated more recently during the population bottleneck. Poirier et al. (2019) found that individuals on Ram Mountain with a translocated parent or grandparent had higher survival and body mass than individuals with only endemic ancestry. This aligns with our findings that admixed sheep tended to have lower genomic inbreeding coefficients.

### 4.2 Limitations of *F*_PED_

By contrast, *F*_PED_ tells a different and largely artefactual story. Pedigree estimates depend on genealogical completeness and cannot reflect inbreeding events that occur outside of the recorded pedigree. Paternity assignments in this population were only possible after the adoption of microsatellite genotyping, leaving inbreeding in early generations effectively concealed from our records. As records accumulate over time, genealogical depth increases, enabling the detection of more consanguineous relationships that improve accuracy but also inflate the inbreeding coefficients of individuals born more recently. This explains the model‐predicted increase in pedigree inbreeding between pre‐ and post‐rescue population groups. It also suggests that a previous study reporting a temporal increase in *F*_PED_ following the 1990s bottleneck (Rioux‐Paquette et al. 2011) was at least partly an artifact of missing ancestral records for early generations. A spurious increase in *F*_PED_ is to be expected in any long‐term study that monitors ancestry, as deeper pedigrees detect more inbreeding (Figure 5). We likely would have drawn the wrong conclusion about the effects of genetic rescue on inbreeding in this population from analyzing *F*_PED_ alone.

Pedigree inbreeding coefficients are poorly suited to tracking inbreeding dynamics over management‐relevant timescales and cannot accurately estimate inbreeding around genetic rescue in wild systems where pedigrees are rarely deep and complete. Pedigrees are still an important component of long‐term wildlife studies, allowing researchers to track multigenerational trends in relatedness, heritability, and reproductive success. As well, the methodological bias over time may be less of a problem when pedigrees are both complete and sufficiently deep to avoid a temporal increase, but this is rare for long‐lived wildlife species where tracking relatedness requires extensive monitoring of behaviour and genetic markers over generations. The Ram Mountain pedigree is more complete than most, as after microsatellite genotyping began in 1987, nearly all gaps have been from immigrant rams and the translocated sheep. Although filtering by depth can mitigate underestimation due to missing ancestral records in wild systems, doing so drastically reduces sample size, introduces a bias toward more successful family lineages and more recent individuals, and cannot account for stochastic inheritance or recombination (Marshall et al. 2002; Kardos et al. 2015). Since high‐density genomic markers remain costly and difficult to obtain for non‐model organisms that may have an imperfect pedigree available (Schmidt et al. 2024), a potential solution is to track admixture over time using a pedigree instead of *F*_PED_, as an increase in the proportion of ancestry from introduced individuals indicates successful integration. This could reveal the effectiveness of genetic rescue while avoiding the noise introduced by the inherent methodological biases of *F*_PED_.

### 4.3 Strengths of *F*_ROH_

The limitations of *F*_PED_ for tracking inbreeding over time in wild systems underscore the value of genomic inbreeding coefficients. Genomic coefficients can theoretically be obtained from a single sampling event without the need for continuous multigenerational monitoring, making them more practical (Wang 2016). They also capture realized inbreeding based on a sample of the genome, enabling higher accuracy (Kardos et al. 2015). ROH‐based genomic inbreeding showed a stronger response to rescue than genome‐wide homozygosity, which remained steady over the study period. Partitioning *F*_ROH_ by ROH size further clarified that intermediate segments (5–20 Mb) reacted strongly to both the population bottleneck and genetic rescue, while shorter segments (1–5 Mb) showed little change and longer segments (> 20 Mb) decreased throughout the study period. Together, these patterns highlight the utility of ROH‐based metrics for resolving fine‐scale inbreeding dynamics and provide a more biologically informative appraisal of the population's genetic health compared to *F*_PED_.

### 4.4 Conservation Implications

Despite the decline following genetic rescue, genomic inbreeding levels in the study population remain elevated. The raw mean *F*_ROH,>1Mb_ of individuals in the post‐rescue group (0.106) is still above the average reported across 78 mammalian populations (0.0745; Brüniche‐Olsen et al. 2018) and exceeds the expectation for first‐cousin offspring (*F* = 0.0625). This suggests that genetic rescue reduced, but did not eliminate, the genomic inbreeding that accumulated from long‐term isolation and the recent bottleneck (Poirier et al. 2019; Deakin et al. 2020). Compared to other wild populations known to have suffered from inbreeding depression, however, the *F*_ROH_ values at Ram Mountain appear modest. For example, the mean value is well below those reported for Florida panthers ( *P. concolor* ) both before (0.63) and following (0.28) genetic rescue (Aguilar‐Gómez et al. 2025), or for isolated populations such as Scandinavian wolves ( *C. lupus* ) (mean = 0.27; Kardos et al. 2017), Soay sheep ( *Ovis aries* ) (mean = 0.24; Stoffel et al. 2021), and Sable Island horses ( *Equus ferus* ) (mean = 0.29; Colpitts et al. 2022). The relatively modest inbreeding levels for Ram Mountain sheep may reflect incomplete isolation, as some nonresident rams immigrate during the rut (Poirier et al. 2019), but this does not eliminate conservation concerns: even relatively moderate *F*_ROH_ values can affect fitness in small, isolated populations (e.g., Kardos et al. 2023), warranting further investigation into how inbreeding is affecting individual fitness.

The Ram Mountain population remains small, isolated, and vulnerable to genomic and extrinsic risks, especially cougar predation (Festa‐Bianchet et al. 2006; Cloutier et al. 2024). Predation can accelerate population decline and increase inbreeding, compounding risks (Armbruster and Reed 2005; Rioux‐Paquette et al. 2011; Kardos et al. 2023). Accurate inbreeding monitoring is thus particularly important where multiple threats intersect, as is often the case in wildlife conservation (Brook et al. 2002; Liao and Reed 2009; Kardos et al. 2023).

## 5 Conclusion

As conservation programs continue to address inbreeding through genetic rescue, incorporating genomic inbreeding monitoring alongside ecological, environmental, and demographic assessment will be essential to evaluate population health and guide adaptive management.

## Funding

This research was funded by the Natural Sciences and Engineering Research Council of Canada (NSERC), the Canada Research Chairs (CRC), the Alberta Conservation Association (ACA), and the University of Western Ontario.

## Conflicts of Interest

The authors declare no conflicts of interest.

## Data Availability

Data used are available on figshare: https://doi.org/10.6084/m9.figshare.31579333.
